# Editorial: Advanced neuroimaging methods in brain disorders, volume II

**DOI:** 10.3389/fnhum.2023.1280306

**Published:** 2023-08-30

**Authors:** Jurong Ding, Dajiang Zhu, Wei Liao

**Affiliations:** ^1^School of Automation and Information Engineering, Sichuan University of Science and Engineering, Zigong, China; ^2^Artificial Intelligence Key Laboratory of Sichuan Province, Sichuan University of Science and Engineering, Zigong, China; ^3^Computer Science and Engineering, University of Texas at Arlington, Arlington, TX, United States; ^4^The Clinical Hospital of Chengdu Brain Science Institute, School of Life Science and Technology, University of Electronic Science and Technology of China, Chengdu, China; ^5^MOE Key Lab for Neuroinformation, High-Field Magnetic Resonance Brain Imaging Key Laboratory of Sichuan Province, University of Electronic Science and Technology of China, Chengdu, China

**Keywords:** neuroimaging, functional magnetic resonance imaging, brain network, machine learning, biomarkers

Using advanced neuroimaging techniques, such as electroencephalogram (EEG), magnetic resonance imaging (MRI) and positron emission tomography (PET), researchers have been working on the mechanisms of human brain under both healthy and different disorders for decades. The development of robust neuroimaging analytical methods will fundamentally advance scientific understanding of the brain and facilitate numerous neuroscience and clinical research studies.

This second volume of the Research Topic continues to provide a forum for researchers who are interested in the recent developments of neuroimaging methods and their applications in various domains. In the end, a total of six papers, including four original research articles, one method and one review, have been collected in this Research Topic. These included papers reported new neuroimaging findings and analytical methods helping to uncover the underlying brain mechanism.

EEG recordings contain vast amounts of physiological and pathological information, which can be useful in the diagnosis and analysis of brain diseases. To address the issue of feature redundancy when extracting EEG features and investigate the importance of patients' age information in EEG detection, Wu et al. proposed a novel lightweight multi-scale aggregation mechanism for EEG pathology detection. They extracted the discriminative multi-scale features by aggregating local and global information, and further integrated these features with patients' age as multimodal features for raw brain signals. The experimental results prove the validity and feasibility of their proposed method. They also verified the significance when incorporating patients' age information in EEG pathology detection.

The utilization of functional MRI (fMRI) technique allows for the investigation of underlying mechanisms of brain function, which has garnered widespread attention from researchers. Two resting-state fMRI (rs-fMRI) studies examined the altered intrinsic brain activity in patients with different disease. By using rs-fMRI and dynamic regional homogeneity (dReHo), Liao et al. investigated the alterations of dynamic intrinsic brain activity among 111 patients with Alzheimer's disease (AD), 29 patients with mild cognitive impairment (MCI) and 73 healthy controls (HC). They reported that patients with AD displayed altered dReHo variability in the right middle frontal gyrus and posterior cingulate gyrus compared to the MCI and HC groups. The dReHo variability in these two brain regions may serve as a potential biomarker for the diagnosis or prognosis of AD. Their study provides a new perspective to uncover the underlying neuropathological mechanisms associated with AD. In the study of Zhu et al., multiple rs-fMRI analyzing methods, including amplitude of low-frequency fluctuation, regional homogeneity, degree centrality and functional connectivity, were used to explore whether resting brain function differs between unaffected parents having Autism spectrum disorder (ASD) children and parents having typically development children. The findings revealed the presence of multiple aberrant brain regions and functional connectivity patterns in unaffected parents having ASD children. Notably, these anomalous rs-fMRI patterns exhibit similarities to the neuroimaging results reported in previous literature on individuals with ASD. Correlation analysis results further reflect that unaffected parents having ASD children may have subclinical elevations in ASD traits. Their study indicates that rs-fMRI metrics could be taken as biomarkers to reveal the neurobiological characteristics of unaffected parents having ASD children.

Structural MRI technique has attracted increasing attention in brain morphometric studies, allowing for the accurate measurement of brain structures, including volume and surface-based morphometric measurements. Using voxel-based morphometry and causal structural covariance network analyses, Zhang et al. evaluated gray matter volume (GMV) alterations and the GMV alterations chronological process in patients with type 2 diabetes (T2D) over the course of their disease. They found that the right temporal pole could be the origin brain region affected by T2D and the GMV alterations in patients with T2D followed a progressive pattern, targeting regions in the limbic-cerebellum-striatal-cortical network. Their work provides a clue to the likely GMV progressive changing pattern of T2D, which may contribute to a better understanding of the progression and an improvement of current diagnosis and intervention strategies for T2D. In order to facilitate researchers to carry out surface-based measurement analysis, Long developed a user-friendly graphical user interface toolbox called “surface-based processing and analysis of MRI” (SPAMRI) based on MATLAB platform, which is compatible with different OS (Windows, Linux and Mac-OS). This toolbox contains pipelines for data preprocessing, statistical analysis, multiple comparison correction, result reporting, extraction of surface measurements and seed-based structural covariance analysis. This toolbox enables a seamless surface-based image processing and MRI dataset analyses, thereby offering convenience to researchers, particularly clinicians and those new to MRI, in their exploration of structural morphologies.

Based on the characteristics and advantages of neuroimaging techniques, a growing number of researchers have been focusing on neuroimaging techniques to conduct research on aphasia. A systematic review from Huang et al. conducted a bibliometric analysis of the neuroimaging research on aphasia to reveal the current hotspots and cutting-edge trends of research in this field. By including 2,799 articles related to the neuroimaging of aphasia from 2004 to 2021, the authors summarized three research hotspots: the dual-stream model of language processing, the mechanism of injury and recovery of post-stroke aphasia, and the diagnostic criteria for primary progressive aphasia variants. They pointed out that future studies should expound the pathological diagnosis of primary progressive aphasia and its relationship with clinical features and MRI imaging by combining with new neuroimaging techniques.

In summary, the second part of this Research Topic presented innovative neuroimaging analytical methods and exciting achievements in clinical applications. These papers contribute to a deeper understanding of the physiological and pathological mechanisms of human brain from a neuroimaging perspective, and also promoting the development of new tools and techniques for processing neuroimaging data. We anticipate that this Research Topic will provide novel insights into neuroscience and clinical research. The advancement of sophisticated neuroimaging techniques and their applications will continue to attract more researchers' attention in the future.

## Author contributions

JD: Conceptualization, Funding acquisition, Investigation, Project administration, Supervision, Writing—original draft, Writing—review and editing. DZ: Conceptualization, Investigation, Project administration, Supervision, Writing—original draft, Writing—review and editing. WL: Conceptualization, Investigation, Project administration, Supervision, Writing—original draft, Writing—review and editing.

